# Knowledge and Determinants of Nonalcoholic Fatty Liver Disease Among Adults in Northern Border Region, Saudi Arabia: A Cross-Sectional Study

**DOI:** 10.3390/diseases14040139

**Published:** 2026-04-09

**Authors:** Yusef Muhana Alenezi, Rana Awad S. Alanazi, Danah Ashwi S. AlShalikhi, Rimas Naif A. Alanazi, Aryam Meshal S. Alanazi, Sarah Ahmed S. Alanazi, Renad Abdulrahman O. Alanazi, Noor Awad S. Alanazi, Baraah Abu Alsel, Fathia Ahmed Mersal, Safya E. Esmaeel, Manal S. Fawzy

**Affiliations:** 1 Department of Family and Community, Faculty of Medicine, Northern Border University, Arar 91431, Saudi Arabia; dr.yusefmd@gmail.com; 2 College of Medicine, Northern Border University, Arar 91431, Saudi Arabia; rana2005awad@gmail.com (R.A.S.A.); dndnmz113@gmail.com (D.A.S.A.); 8remax09@gmail.com (R.N.A.A.); aryamm461@icloud.com (A.M.S.A.); sarh22410@gmail.com (S.A.S.A.); irenad1436@gmail.com (R.A.O.A.); na.1887788@gmail.com (N.A.S.A.); 3 Medical Sciences & Preparatory Year Department, North Private College of Nursing, Arar 73244, Saudi Arabia; baraahsel@nec.edu.sa; 4 Public Health Nursing Department, College of Nursing, Northern Border University, Arar 91431, Saudi Arabia; fathia.hassan@nbu.edu.sa; 5 Department of Physiology, College of Medicine, Northern Border University, Arar 91431, Saudi Arabia; safya.ebraheem@nbu.edu.sa; 6 Center for Health Research, Northern Border University, Arar 73213, Saudi Arabia

**Keywords:** nonalcoholic fatty liver disease, NAFLD, knowledge, health education, risk factors, adults, Saudi Arabia

## Abstract

Background/Objectives: Nonalcoholic fatty liver disease (NAFLD), also referred to as metabolic dysfunction-associated steatotic liver disease (MASLD), affects roughly one-quarter of the global population and represents a major public health concern. Despite its rising prevalence and potential for serious complications, NAFLD remains underrecognized and poorly understood in many communities. This study aimed to assess knowledge of NAFLD and its determinants among adults in the Northern Border Region of Saudi Arabia. Methods: A descriptive, population-based cross-sectional study was conducted using a previously validated online questionnaire adapted from published NAFLD awareness instruments, administered to adults residing in the Northern Border Region of Saudi Arabia. Data were analyzed using Python (statsmodels, version 0.14), and non-parametric tests, correlation analyses, and multivariable linear regression were used to examine NAFLD knowledge and its associated determinants. Results: A total of 1016 adults (mean age 34.7 ± 11.8 years) were included in the analysis. The mean NAFLD knowledge score was 14.6 ± 8.3 out of 30 (48.7% correct responses), with a median of 16 (interquartile range 8–21). Overall, 59.2% of participants had poor knowledge, 26.8% had moderate knowledge, and 14% had good knowledge. In bivariate analyses, educational level (χ^2^ = 15.62, *p* < 0.001), family history of liver disease (*p* = 0.001), body weight category (*p* = 0.003), and smoking status (*p* = 0.007) were significantly associated with NAFLD knowledge. In multivariable linear regression, university education (B = 2.783, 95% CI 0.627–4.940, *p* = 0.011) was an independent positive predictor of higher knowledge scores. Current smoking (B = −1.857, 95% CI −3.477 to −0.237, *p* = 0.025), private-sector employment (B = −1.934, 95% CI −3.867 to −0.001, *p* = 0.050), and overweight status (B = −4.119, 95% CI −7.337 to −0.901, *p* = 0.012) were independently associated with lower knowledge scores. The final model explained 2.2% of the variance in knowledge (adjusted R^2^ = 0.022). Conclusions: This study demonstrates generally low levels of NAFLD knowledge among adults in the Northern Border Region of Saudi Arabia, with only a minority achieving good knowledge scores. The findings underscore the need for targeted health promotion initiatives, educational interventions, and public campaigns to improve awareness of NAFLD and to support its prevention and management.

## 1. Introduction

Nonalcoholic fatty liver disease is characterized by hepatic steatosis in individuals who do not consume alcohol at hepatotoxic doses, typically presenting as macrovesicular fat accumulation with minimal or no lobular inflammation [1]. It encompasses a spectrum ranging from nonalcoholic fatty liver (simple steatosis) to nonalcoholic steatohepatitis, which may progress to fibrosis, cirrhosis, and hepatocellular carcinoma [2]. NAFLD has become one of the leading causes of chronic liver disease worldwide, with recent reports estimating a prevalence of roughly 25–30% in adults and more than one billion affected individuals globally [3,4]. Emerging data also indicate a shift toward younger age groups, with increasing NAFLD burden reported among adolescents and young adults, which is projected to translate into a substantial future disease burden [4].

Beyond liver-related morbidity, NAFLD is closely linked to extrahepatic complications and is now recognized as a multisystem disease [5]. It is strongly associated with obesity, insulin resistance, metabolic syndrome, type 2 diabetes mellitus, cardiovascular disease, and chronic kidney disease [6]. It has been implicated as an etiological factor in hepatocellular carcinoma even in the absence of cirrhosis [7]. The rising prevalence of NAFLD parallels global increases in obesity, unhealthy dietary patterns, and sedentary lifestyles, making it a major contributor to the overall burden of metabolic disease [8]. Given its close association with cardiometabolic risk, an expert consensus recently proposed renaming NAFLD as metabolic dysfunction-associated steatotic liver disease (MASLD), although the term NAFLD remains widely used in clinical practice and public health research and is adopted in the present study for consistency [9].

In the Middle East, NAFLD prevalence has risen markedly over recent decades, driven largely by high rates of obesity and type 2 diabetes [10]. In Saudi Arabia, modeling and epidemiological studies suggest that approximately one-quarter to one-third of adults may have NAFLD, with projections indicating further growth in the coming years [11,12,13]. This pattern is particularly concerning, given the existing high burden of metabolic disorders, the anticipated increase in advanced liver disease, and the associated implications for healthcare resource utilization [5]. Notably, the Northern Border Region carries a particularly high burden of metabolic risk factors; studies from the region report elevated rates of diabetes, obesity, and cardiovascular comorbidities, with diabetes control rates among the lowest nationally, underscoring the heightened vulnerability of this population to NAFLD and its complications [14,15]. Despite this regional risk profile, no dedicated epidemiological data on NAFLD prevalence in this region are currently available, highlighting a critical evidence gap that warrants focused investigation.

At the national level, while Saudi Arabia has implemented broad preventive health initiatives under Vision 2030, including the “Taakad” screening program and Ministry of Health campaigns targeting metabolic and liver disease, these efforts have focused primarily on viral hepatitis and general non-communicable disease prevention, with limited specific public awareness programming directed at NAFLD [11,16]. Consequently, low community knowledge of NAFLD may reflect not only personal neglect but also limited organized exposure to NAFLD-specific health education [17].

Despite this growing burden, studies from different settings have shown that public awareness of NAFLD, its risk factors, consequences, and diagnostic approaches, remains limited, even among high-risk groups [18]. Many individuals are unaware of NAFLD as a specific condition, underestimate their personal risk, or do not recognize the role of lifestyle modification in preventing progression [19]. In Saudi Arabia, most available research has focused on clinical or high-risk populations, such as patients with diabetes or obesity. In contrast, fewer studies have examined knowledge of NAFLD in the general adult population, and data from the Northern Border Region are particularly scarce [20]. Understanding how much the public knows about NAFLD and which sociodemographic factors shape this knowledge is essential for designing effective, regionally tailored health education strategies. Therefore, the present study aimed to assess knowledge levels about NAFLD and identify its determinants among adults in the Northern Border Region of Saudi Arabia.

## 2. Materials and Methods

### 2.1. Participants and Study Design

A cross-sectional study was conducted to assess knowledge of and the determinants of NAFLD among adults residing in the Northern Border Region of Saudi Arabia between 10 December 2025 and 10 January 2026. The Northern Border Region was selected for several reasons. First, the region carries a disproportionately high burden of metabolic risk factors, including obesity, type 2 diabetes, and cardiovascular disease, that are closely linked to NAFLD development, yet remains among the least studied regions in Saudi Arabia with respect to liver disease awareness [14]. Second, no prior community-based data on knowledge of NAFLD are available from this region, representing a critical evidence gap. Third, the region’s distinct demographic profile, characterized by a relatively young age structure and the presence of Northern Border University, provides a unique opportunity to examine knowledge determinants across diverse educational and occupational groups. Eligible participants were men and women aged 18 years and older who were residents of the Northern Border Region. Individuals younger than 18 years and adults who were not residents of the Northern Border Region were excluded from participation. Additional exclusion criteria included failure to provide electronic informed consent and submission of incomplete questionnaire responses (defined as missing data on one or more knowledge items).

### 2.2. Sample Technique and Sample Size

Sample size was calculated for this cross-sectional survey using the single-proportion formula:

$$n=\frac{Z_{{\alpha /}_{2}}^{2}\cdot p(1-p)}{d^{2}}$$

assuming a 50% expected proportion of adequate NAFLD knowledge (maximum variability), a 95% confidence level (*Z* = 1.96), and a 3.2% margin of error (*d* = 0.032); this margin was selected to achieve a sample size comfortably exceeding 900 participants within the logistical constraints of a single-region convenience survey, while remaining within the conventionally accepted range of 3–5% for community-based health knowledge studies. This yielded a minimum required sample of approximately 945 participants. Allowing for an anticipated non-response rate of about 10% and incomplete data, the target sample size was inflated to roughly 1050 adults. Ultimately, 1016 participants provided complete data and were included in the final analysis. A non-probability convenience sampling approach was used, and all participants provided electronic informed consent before completing the survey. Participants were approached through community groups, regional networks, and the research team’s personal contacts active in the region.

It is acknowledged that convenience sampling limits the generalizability of findings to the broader Northern Border Region population. Participants were recruited from accessible community settings, and the resulting sample may over-represent individuals with higher educational attainment and greater health engagement. These limitations are discussed further in Section 4.5.

### 2.3. Data Collection Tool

Data were collected using an online questionnaire previously validated to assess NAFLD awareness and related constructs [18,21]. The questionnaire comprised four sections (Supplementary File S1):

(a) Sociodemographic characteristics (10 items), including age, sex, weight, height, marital status, occupation, educational level, history of chronic disease, history of NAFLD or other liver disease, smoking status, and place of residence.
(b) General knowledge about NAFLD (11 items), covering causes, prevalence in Saudi Arabia, common symptoms, and potential complications; response options were “yes”, “no”, or “don’t know”.
(c) Knowledge of determinants and risk factors for developing fatty liver (13 items), with the same three response options.
(d) Knowledge related to prevention and management of NAFLD (6 items), again answered with “yes”, “no”, or “don’t know”.

The questionnaire was prepared in Arabic, disseminated via social media platforms (Facebook and WhatsApp), and subsequently translated back into English for reporting. Content validity was evaluated by a panel of internal medicine specialists, who reviewed the items for clarity, relevance, and coverage of key domains. A pilot test was conducted with 30 adults from the Northern Border Region to assess comprehensibility and feasibility; the pilot data were not included in the final analysis [20].

### 2.4. Scoring of NAFLD Knowledge

Knowledge of NAFLD was quantified using 30 items covering general information, risk factors, and prevention/management. Each item was scored dichotomously, with 1 point for a correct answer and 0 for an incorrect or “don’t know” response. Equal weighting was applied on the basis that all items assess factual knowledge within a single construct and were judged by the content validity panel to be of comparable importance; this approach is standard in community health knowledge surveys and facilitates comparability with published studies using identical or similar instruments [18,21]. Incorrect responses and “don’t know” responses were combined into a single non-correct category because both reflect the absence of verified knowledge, which is the construct of interest; this conservative approach avoids rewarding guessing and is consistent with precedent in NAFLD knowledge measurement. Individual item scores were summed to generate a total knowledge score ranging from 0 to 30, with higher scores indicating greater knowledge. For ease of interpretation, the total score was converted to a percentage using the formula: (participant score/30) × 100. Percentage scores were then categorized according to Bloom’s cutoffs into poor (≤59%), moderate (60–79%), and good (80–100%) knowledge; these thresholds were pre-specified and applied uniformly across analyses. It is acknowledged that Bloom’s cutoffs were originally developed for educational achievement testing; their application here is pragmatic and consistent with their use in comparable public health knowledge surveys conducted in Saudi Arabia and other areas [22,23,24,25], and the resulting categories should therefore be interpreted as approximate descriptive benchmarks rather than psychometrically validated classifications. Internal consistency of the knowledge scale was high, with a Cronbach’s alpha of 0.935 for the 30 items. Subdomain reliability was similarly strong: α = 0.891 for the general knowledge subdomain (11 items), α = 0.887 for the risk factors subdomain (13 items), and α = 0.832 for the prevention and management subdomain (6 items). The high overall α should be interpreted in the context of scale length, as Cronbach’s α is sensitive to the number of items; the consistently strong subdomain alphas nonetheless support the internal coherence of each construct independently.

### 2.5. Statistical Analysis

Continuous variables were examined for normality using the Shapiro–Wilk test and visual inspection of histograms and Q–Q plots. Variables approximating a normal distribution were summarized as mean ± standard deviation (SD), whereas skewed variables were summarized as median and interquartile range (IQR). Categorical variables were reported as frequencies and percentages. Because NAFLD knowledge scores deviated significantly from normality, non-parametric tests were used for bivariate analyses. Bivariate associations between NAFLD knowledge scores and categorical predictors were assessed using the Mann–Whitney U test for two-level variables and the Kruskal–Wallis test for variables with more than two categories. For each bivariate comparison, effect sizes were calculated (epsilon-squared for Kruskal–Wallis tests and rank-biserial correlation for Mann–Whitney U tests) to complement *p* values. Associations between categorical variables were evaluated using chi-square tests, with Cramér’s V reported where appropriate as a measure of association strength.

To identify independent predictors of NAFLD knowledge, multivariable linear regression analysis was performed. Specifically, multiple linear regression (MLR) was employed to model the linear relationship between a single continuous dependent variable (total NAFLD knowledge score) and multiple independent predictors. The term ‘multivariable’ is used throughout, in accordance with epidemiological convention, to distinguish analyses involving multiple predictors from univariable or bivariate analyses [26]. Candidate variables were selected a priori based on clinical relevance and previous literature on NAFLD awareness and health literacy. All selected variables were entered simultaneously into the initial model, and model refinement emphasized parsimony using information criteria, including the Akaike Information Criterion (AIC) and Bayesian Information Criterion (BIC). Regression results are presented as unstandardized beta coefficients (β) with 95% confidence intervals. Interaction terms between educational level and age, and between educational level and employment status, were tested and retained only if statistically significant.

Model assumptions were evaluated through standard diagnostic procedures. Multicollinearity was assessed using variance inflation factors (VIF), with values <10 considered acceptable. Residual distributions were examined using the Shapiro–Wilk test and Q–Q plots to assess normality, while the Breusch–Pagan test was used to evaluate homoscedasticity. Minor deviations from ideal assumptions were judged acceptable given the large sample size and the robustness of linear regression under asymptotic conditions. Specifically, although the knowledge score deviated from normality in bivariate analyses, multivariable linear regression was retained as the primary analytical approach because the central limit theorem supports the asymptotic normality of parameter estimates in samples of this size (n = 1016), and because linear regression provides interpretable effect estimates (unstandardized β coefficients) that are directly comparable across predictors and with similar published studies. Where the Breusch–Pagan test indicated heteroscedasticity, heteroscedasticity-consistent (robust) standard errors were applied to ensure valid inference; this adjustment is reflected in the reported confidence intervals and *p*-values. Statistical significance was set at a two-sided *p*-value ≤ 0.05. All analyses were conducted using Python (statsmodels, version 0.14).

### 2.6. Ethical Consideration

The study adhered to national and institutional ethical guidelines for research involving human participants. Ethical approval was obtained from the “Local Bioethics Committee of Northern Border University (HAP-09-A-043; decision number 97-25-H) on 8 December 2025.” Before accessing the questionnaire, all potential participants were provided with detailed information about the study objectives, procedures, potential benefits, and any foreseeable risks, and electronic informed consent was obtained. Participation was entirely voluntary, and participants were informed of their right to withdraw at any time without penalty. All data were handled confidentially and stored securely, with access restricted to the research team.

## 3. Results

### 3.1. Participant Characteristics and Overall Knowledge

**Figure 1:**
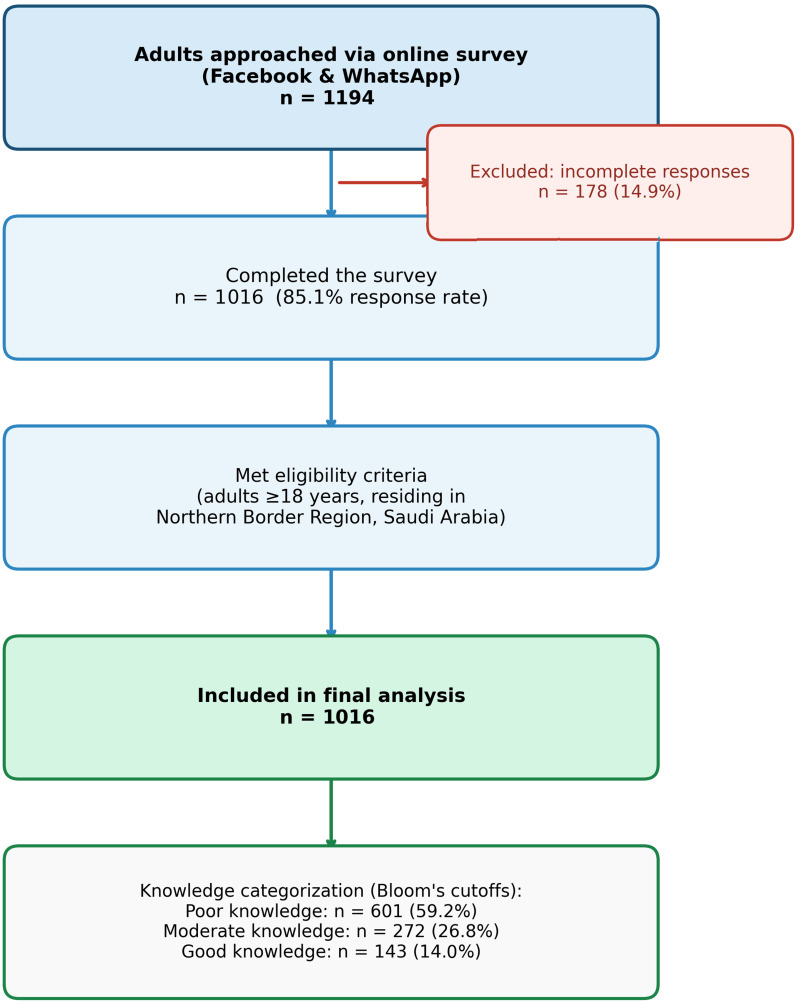
Study participant flow diagram.

Of the 1194 individuals who initiated the survey, 1016 (85.1%) provided complete data and were included in the analysis (Figure 1). The mean age of participants was 34.7 ± 11.8 years, 65.5% had attained university-level education, and just over half were married (57.4%) and not working or retired (57.4%). Chronic diseases were reported by 19.7% of respondents, 25.3% were current or former smokers, and 7.1% reported a family history of liver disease (Table 1).

**Figure 2:**
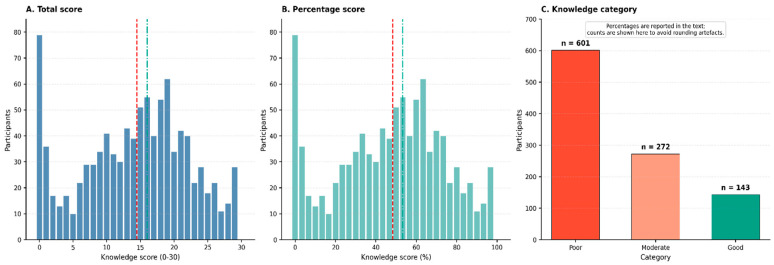
Distribution and categorization of NAFLD knowledge scores. (**A**) the total score distribution, (**B**) the percentage scores (with red and green dashed lines indicating the mean and median values, respectively), and (**C**) the knowledge categories based on predefined Bloom cutoffs.

The mean NAFLD knowledge score was 14.6 ± 8.3 out of a possible 30, corresponding to a mean percentage score of 48.7% (Table 1). Knowledge scores deviated significantly from normality (Shapiro–Wilk W = 0.960, *p* < 0.001), with a median of 16 (interquartile range [IQR] 8–21) and a negatively skewed distribution (Figure 2A). Visual inspection of Figure 2A revealed a high concentration of observations at low score values, raising the possibility of zero inflation or subpopulation heterogeneity. To address this, the raw data were verified to confirm that zero and near-zero scores reflected genuine participant responses rather than coding errors, missing-value imputation, or data-entry artifacts; no such anomalies were identified. Participants who scored zero did not differ systematically from the remainder of the sample in terms of age, sex, or educational level, suggesting that extreme low scores reflect a genuine stratum of very limited NAFLD knowledge rather than a data quality issue.

When expressed as percentages, the median knowledge score was 53.3% (Figure 2B). Using predefined cutoffs, 59.2% of participants were categorized as having poor knowledge, 26.8% as having moderate knowledge, and 14% as having good knowledge (Figure 2C).

### 3.2. Bivariate Associations with NAFLD Knowledge

**Figure 3:**
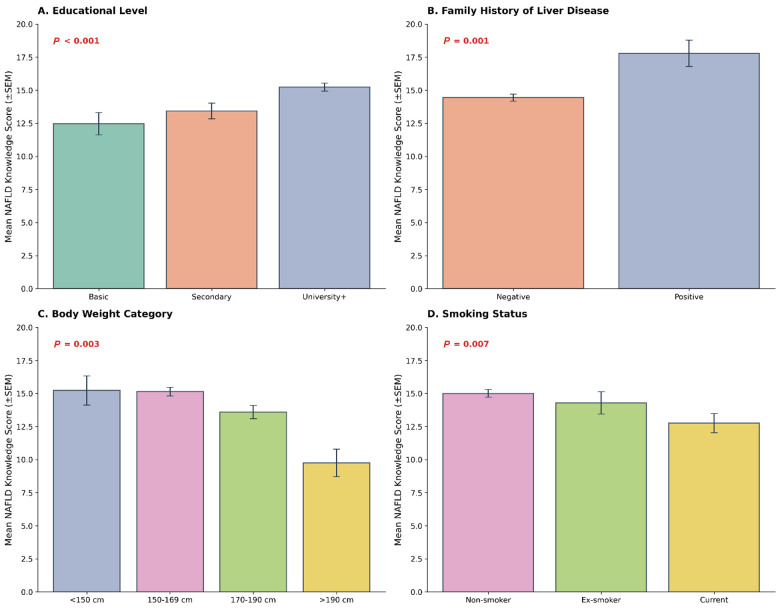
Significant bivariate associations with NAFLD knowledge scores. Boxplots of NAFLD knowledge scores stratified by (**A**) educational level, (**B**) Family history of liver diseases, (**C**) Body weight, and (**D**) smoking status. Horizontal lines show medians, boxes represent interquartile ranges, whiskers extend to 1.5 × IQR, and dots indicate outliers. *p* values correspond to Kruskal–Wallis or Mann–Whitney U tests, as appropriate.

Bivariate analysis was conducted to identify sociodemographic and clinical factors associated with NAFLD knowledge scores (Table 2 and Figure 3). Four variables demonstrated statistically significant associations with NAFLD knowledge scores. Educational level was a significant determinant of NAFLD knowledge (χ^2^ = 15.62, *p* < 0.001, η^2^ = 0.015), with a clear gradient in knowledge across educational strata. Participants with university education recorded the highest median knowledge scores (median 16, IQR 9–21), followed by those with secondary education (median 15, IQR 6–20), while those with basic education recorded the lowest scores (median 12, IQR 7–16.8).

Furthermore, family history of liver disease was also significantly associated with NAFLD knowledge levels (U = 41,286, *p* = 0.001, r = 0.231). Body weight category showed a significant association with knowledge scores (χ^2^ = 13.72, *p* = 0.003, η^2^ = 0.014), with normal-weight participants recording higher median scores (median 16, IQR 8–21) compared to overweight (median 15, IQR 8–21) and obese participants (median 15, IQR 7–20).

Smoking status was significantly associated with NAFLD knowledge (U = 47,334, *p* = 0.007, r = 0.150), with never-smokers reporting higher median scores (16, IQR 8–21) than current or former smokers (14, IQR 7–20).

All remaining variables: age group, sex, marital status, employment status, and chronic disease history, did not demonstrate significant associations with NAFLD knowledge scores, with negligible effect sizes throughout (Table 2).

### 3.3. Multivariable Predictors of NAFLD Knowledge

A multivariable linear regression model was constructed to identify independent predictors of NAFLD knowledge scores (Table 3). The overall model was statistically significant (F = 2.751, *p* = 0.001, adjusted R^2^ = 0.022). Interaction terms between educational level and age (F = 1.24, *p* = 0.291) and between educational level and employment status (F = 0.87, *p* = 0.483) were tested. Still, they did not reach statistical significance and were therefore excluded from the final model.

University education was the only significant positive independent predictor, with university-educated participants scoring 2.783 points higher than those with basic education (B = 2.783, SE = 1.099, β = 0.150, 95% CI: 0.627 to 4.940, *p* = 0.011). Secondary education did not independently predict knowledge scores (B = 0.959, SE = 1.180, β = 0.047, 95% CI: −1.357 to 3.276, *p* = 0.417).

Three variables were significant negative independent predictors. Overweight status was the strongest negative predictor, with overweight participants scoring 4.119 points lower than those of normal weight (B = −4.119, SE = 1.640, β = −0.080, 95% CI: −7.337 to −0.901, *p* = 0.012). Current smoking was also a significant negative predictor, with current smokers scoring 1.857 points lower than non-smokers (B = −1.857, SE = 0.826, β = −0.073, 95% CI: −3.477 to −0.237, *p* = 0.025). Private-sector employment was associated with significantly lower knowledge scores than not working or being retired (B = −1.934, SE = 0.985, β = −0.065, 95% CI: −3.867 to −0.001, *p* = 0.050).

All remaining variables, age, sex, secondary education, governmental employment, previous smoking, family history of liver disease, history of chronic disease, and obese or underweight status, did not reach statistical significance in the multivariable model (all *p* > 0.05).

### 3.4. Item-Level Knowledge Patterns

**Figure 4:**
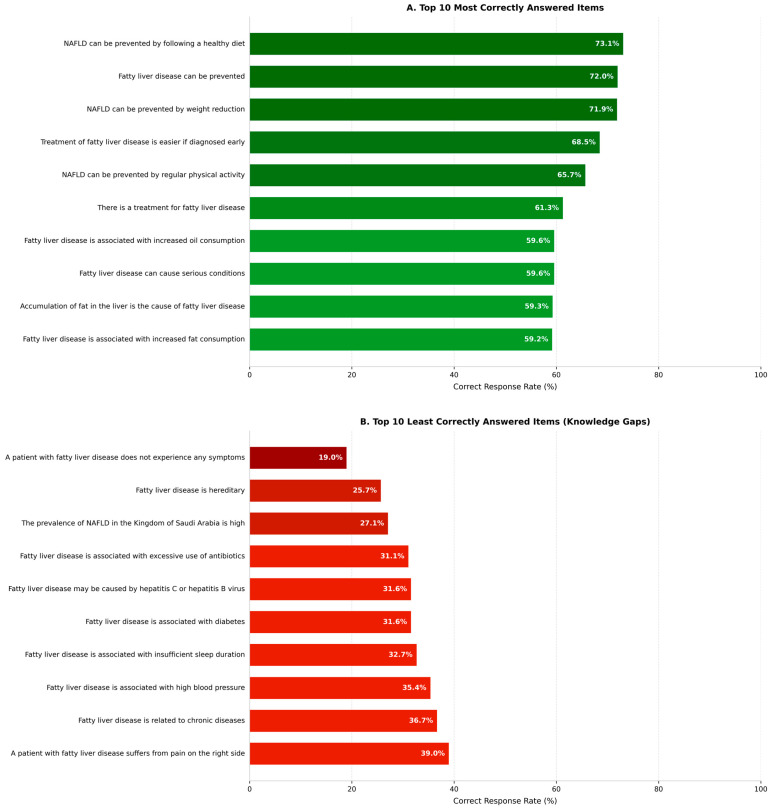
Item-level performance on the NAFLD knowledge questionnaire (N = 1016). (**A**) The 10 items with the highest correct response rates. (**B**) The 10 items with the lowest correct response rates represent the primary knowledge gaps in this population. Bars represent the percentage of participants providing a correct response to each item. Items were drawn from three domains: general knowledge about NAFLD, risk factor awareness, and knowledge of prevention and management.

Item-level analysis revealed substantial heterogeneity in knowledge of NAFLD (Figure 4). The highest correct response proportions were observed for items related to prevention and lifestyle modification, including healthy diet (73.1%) and weight loss (71.9%). In contrast, the lowest correct response rates were observed for items related to symptomatology, epidemiology, and comorbid conditions, such as asymptomatic presentation (19.0%), hereditary risk (25.7%), and national prevalence estimates (27.1%). These patterns highlight specific content areas where public knowledge is particularly limited.

### 3.5. Model Diagnostics

To evaluate the robustness of the multivariable regression model, comprehensive diagnostic checks confirmed that the model’s assumptions were adequately met (Table 4). Variance inflation factors ranged from 1.02 to 3.66, all below the threshold of 10, indicating acceptable multicollinearity. The Shapiro–Wilk test indicated non-normal residual distribution (W = 0.977, *p* < 0.001), and the Breusch–Pagan test indicated heteroscedasticity. The residual mean was zero, indicating no systematic bias. The adjusted R^2^ of the final model was 0.022.

## 4. Discussion

The present study investigated general knowledge of NAFLD, as well as determinants and knowledge related to prevention and management, among adults in the Northern Border Region of Saudi Arabia. A substantial proportion of participants (59.2%) were classified as having poor knowledge, indicating marked gaps in awareness of NAFLD in this community. Comparable surveys from other regions of Saudi Arabia have similarly reported low to fair levels of knowledge about NAFLD, reinforcing the concern that public understanding of this condition remains inadequate at the national level [20,27,28,29,30].

Consistent with our findings, several studies from Jeddah, Taif, Hail, Jazan, and the Riyadh region have documented limited awareness, with only a minority of respondents achieving good knowledge scores or demonstrating accurate understanding of NAFLD risk factors and management [20,27,28,29,30]. International data point in the same direction: studies from the United States, China, and Egypt have reported that many adults are unaware of NAFLD as a diagnosis, have never heard of the condition, or possess fair to moderate knowledge [21,31,32]. Notably, one study from Jazan reported higher levels of awareness, with more than half of participants classified as having good knowledge, suggesting that local context, sampling frames, or prior exposure to liver disease campaigns may influence observed levels of knowledge [18].

### 4.1. Sociodemographic Determinants of NAFLD Knowledge

Educational attainment emerged as a key determinant of knowledge about NAFLD in this study, with university-educated participants scoring significantly higher than those with basic or secondary education. This aligns with previous research from Saudi Arabia and elsewhere showing that higher education is positively associated with health knowledge and literacy, including awareness of NAFLD and other chronic diseases [2,28]. These findings underscore the importance of tailoring educational interventions to individuals with lower levels of education, who may be less likely to access or interpret health information effectively [13].

In contrast, age group, sex, marital status, employment status, and chronic disease history did not demonstrate significant associations with NAFLD knowledge in either bivariate or multivariable analyses. Body weight category was significantly associated with knowledge at the bivariate level (*p* = 0.003); notably, overweight status emerged as a significant independent negative predictor in the multivariable model (B = −4.119, *p* = 0.012), suggesting that overweight individuals may have lower engagement with metabolic health information despite being at elevated risk. Similar patterns have been reported in other NAFLD awareness studies, where occupation and age did not consistently predict knowledge levels [18,20]. This suggests that limited NAFLD awareness is pervasive across demographic strata and is not confined to specific age or occupational groups [28]. However, some studies have documented variation by age, gender, and employment in other settings [33]. The lack of association with chronic disease status also indicates that having comorbid conditions does not necessarily translate into greater awareness of NAFLD, highlighting potential missed opportunities for counseling and patient education in clinical encounters [34].

### 4.2. Smoking, Overweight, and Employment as Knowledge Determinants

Family history of liver disease was significantly associated with NAFLD knowledge at the bivariate level (U = 41,286, *p* = 0.001, r = 0.231), suggesting that individuals with affected family members may receive more information about liver conditions, perceive themselves as being at higher risk, or pay greater attention to liver-related health messages. However, this association did not retain statistical significance in the multivariable model (B = −0.170, *p* = 0.868) after adjustment for sociodemographic covariates, indicating that the bivariate association was largely attributable to confounding by correlated variables such as educational level and employment status. Such individuals may nonetheless represent an important target group for more intensive counseling and family-based education interventions [13].

Conversely, current or former smokers had significantly lower NAFLD knowledge scores, independent of other sociodemographic and health-related factors. While the present study was not designed to examine causal pathways, this finding may reflect broader lifestyle and health literacy patterns among smokers, who, in previous research, have been shown to engage less with preventive health information [35]. Given that smoking is associated with an increased risk of NAFLD-related hepatic fibrosis and adverse liver outcomes [36], this knowledge deficit is particularly concerning and supports the integration of NAFLD education into smoking cessation and cardiovascular risk-reduction programs [37].

### 4.3. Model Performance and Unexplained Variance

The multivariable model explained a modest proportion of variance in NAFLD knowledge (adjusted R^2^ = 0.022). This magnitude is comparable to that reported in many population-based studies of health knowledge and literacy, in which sociodemographic variables often account for only a small fraction of individual variability [38,39]. However, the low explained variance is not simply a methodological limitation to be acknowledged and set aside; it carries substantive implications for how NAFLD knowledge is distributed in this population and for what future research must measure to understand it better. Several constructs not captured in the present survey are likely responsible for a substantial portion of the unexplained variance. First, prior exposure to dedicated liver disease health campaigns is a plausible and proximate determinant. Saudi Arabia has conducted national and regional campaigns targeting metabolic diseases, diabetes, and obesity, but dedicated public awareness initiatives specifically addressing NAFLD remain limited in the Northern Border Region [14,40,41,42,43]. Individuals who have encountered targeted liver health messaging, whether through primary care encounters, community health fairs, or social media campaigns, would be expected to score considerably higher regardless of their educational or employment background, explaining variance that sociodemographic variables alone cannot capture. Second, the quality and content of clinician communication are likely major unmeasured drivers. Patients who have received explicit counseling about fatty liver disease from a physician, for instance, during management of obesity, diabetes, or dyslipidemia, have a direct informational pathway to NAFLD knowledge that operates independently of formal education [44]. Future studies should include structured items on whether a healthcare provider has ever informed participants about fatty liver disease and the depth of that communication. Third, functional health literacy, the capacity to obtain, process, and act on health information, is conceptually distinct from educational attainment and is not fully captured by years of schooling alone [45]. Validated Arabic-language health literacy instruments would be needed to capture this dimension and likely explain a meaningful increment of variance beyond what educational level alone accounts for. Fourth, media consumption habits and digital health information-seeking behavior represent a rapidly growing source of health knowledge in Saudi Arabia, where smartphone penetration and social media use are among the highest globally [46]. Whether a participant regularly engages with health-related content on digital platforms can substantially influence NAFLD awareness, independent of formal education or employment status. Fifth, cultural and familial norms around health disclosure and illness perception may modulate how individuals in this region engage with preventive health information [47]. In contexts where liver disease may carry social stigma or cultural associations with alcohol use, which is prohibited in Saudi Arabia, individuals may be less likely to seek or retain information about hepatic conditions regardless of their sociodemographic profile [48].

The primary aim of the regression analysis was explanatory rather than predictive: to identify independent correlates of NAFLD knowledge while adjusting for plausible confounders. In such contexts, low R^2^ values are expected and do not undermine the relevance of statistically significant associations, which can still provide meaningful insight into determinants of health knowledge. The robust associations with university education, current smoking, overweight status, and private-sector employment, despite the low overall explained variance, support the interpretation that these factors exert genuine, albeit modest, influences on NAFLD knowledge in this population. Future studies aiming to build more explanatory models should incorporate validated measures of health literacy, structured assessment of prior NAFLD-specific clinician counseling, digital health information-seeking behavior, and instruments capturing illness perception to more fully characterize the determinants of NAFLD awareness in this region.

### 4.4. Implications for Practice and Policy

Taken together, the findings highlight an urgent need for targeted health promotion efforts to raise awareness of NAFLD in the Northern Border Region and, potentially, in other Saudi regions with similar profiles. Public health campaigns should emphasize early detection, lifestyle-related risk factors, and the potential reversibility of early-stage NAFLD through diet, weight management, and physical activity. Interventions could be delivered through primary care centers, community health programs, digital platforms, and mass media, with special emphasis on individuals with lower educational attainment, smokers, and those without a known family history of liver disease, who may not recognize their risk.

Moreover, integrating NAFLD education into routine clinical practice, particularly in clinics managing obesity, diabetes, and cardiovascular disease, could help bridge current knowledge gaps. Training healthcare providers to deliver clear, culturally appropriate messages about NAFLD and its prevention may enhance patient understanding and support behavior change. Partnerships among health authorities, educational institutions, and media organizations could also facilitate sustained, regionally tailored campaigns that normalize discussions of metabolic health and liver disease.

### 4.5. Study Limitations

Several limitations should be considered when interpreting these results. First, the modest adjusted R^2^ indicates that most of the variability in NAFLD knowledge remains unexplained, likely due to the omission of key determinants such as exposure to health information, the quality of physician counseling, media use patterns, and psychosocial characteristics. Second, the cross-sectional design precludes causal inference; observed associations should therefore be interpreted as correlational. Third, knowledge was assessed using a self-administered questionnaire, which may be subject to social desirability and recall biases and may not fully capture applied or functional knowledge in real-life settings. Furthermore, the questionnaire did not capture information on alcohol consumption history, prior NAFLD diagnosis, or previous testing for liver disease. Although the absence of significant alcohol intake defines NAFLD, the absence of formal alcohol screening means that a small proportion of participants with hazardous alcohol use cannot be entirely excluded. Future studies should incorporate validated alcohol use screening tools and document prior NAFLD testing status to allow more nuanced interpretation of knowledge levels across risk strata. Additionally, the high internal consistency of the knowledge questionnaire (Cronbach’s α = 0.935) suggests potential item redundancy, which may have introduced construct overlap and attenuated the scale’s discriminative capacity; future studies should consider item reduction or confirmatory factor analysis to optimize the scale’s structure.

Finally, the study was conducted in a single region of Saudi Arabia using a convenience sample. The educational distribution of the sample, with 65.5% of participants holding university-level qualifications, warrants specific acknowledgment, given that university education was identified as a significant independent predictor of NAFLD knowledge. A sample enriched for university graduates may yield higher mean knowledge scores than would be observed in a fully representative population sample, and the reported knowledge levels should therefore be interpreted with appropriate caution. Notably, this educational profile is consistent with those reported in previous community-based studies conducted in the Northern Border Region [49,50], suggesting it reflects the actual demographic characteristics of the accessible adult population in this area rather than a systematic recruitment bias, attributable in part to the region’s young age structure, the presence of Northern Border University, and predominantly public-sector employment patterns. Future studies employing stratified or probability sampling with explicit educational quotas would provide more precise population-level estimates of NAFLD knowledge in this region and beyond.

## 5. Conclusions

This study demonstrated generally poor knowledge of NAFLD and its determinants among adults in the Northern Border Region of Saudi Arabia. Higher educational attainment was independently associated with better NAFLD knowledge, whereas current smoking, overweight status, and private-sector employment were independently associated with lower knowledge scores. At the bivariate level, family history of liver disease, body weight category, and smoking status also demonstrated significant associations, underscoring the multifactorial nature of NAFLD awareness. These findings highlight the need for tailored health promotion strategies, educational interventions, and public campaigns to raise awareness of NAFLD, particularly among sociodemographic groups with low knowledge. Strengthening public and professional awareness of NAFLD and its consequences is essential to support earlier detection, facilitate lifestyle-based prevention, and mitigate the growing burden of fatty liver disease. It should be noted that NAFLD, as used throughout this study, is synonymous with the recently adopted term MASLD; future studies in this region should adopt the updated nomenclature to facilitate comparability with the emerging global literature.

## Figures and Tables

**Table 1:** Participant characteristics and NAFLD knowledge scores (N = 1016).

Characteristic	*n* (%) or Mean ± SD	NAFLD Knowledge Score *
**Age (years)**	34.7 ± 11.8	—
≤29	356 (35.0)	15 (8–21)
30–44	441 (43.4)	16 (8–21)
≥45	219 (21.6)	15 (7–20)
**Sex**		
Male	487 (47.9)	16 (8–21)
Female	529 (52.1)	15 (8–21)
**Educational level**		
Basic	86 (8.5)	12 (7–16.8)
Secondary	265 (26.1)	15 (6–20)
University	665 (65.5)	16 (9–21)
**Marital status**		
Single	433 (42.6)	15 (8–21)
Married	583 (57.4)	16 (8–21)
**Employment status**		
Not working/retired	584 (57.4)	16 (8–21)
Government sector	211 (20.8)	16 (9–22)
Private sector	221 (21.8)	14 (7–20)
**Body weight category**		
Normal	372 (36.6)	16 (8–21)
Overweight	337 (33.2)	15 (8–21)
Obese	307 (30.2)	15 (7–20)
**Chronic disease**		
No	816 (80.3)	15 (8–21)
Yes	200 (19.7)	16 (8–21)
**Smoking status**		
Never	759 (74.7)	16 (8–21)
Current/former	257 (25.3)	14 (7–20)
**Family history of liver disease**		
No	944 (92.9)	16 (8–21)
Yes	72 (7.1)	16 (11–22)
**Total NAFLD knowledge score (0–30)**	14.6 ± 8.3	16 (8–21)

* Knowledge scores are presented as median (interquartile range). —: Not applicable.

**Table 2:** Bivariate associations between participant characteristics and NAFLD knowledge score.

Variable	Statistical Test	Test Statistic	*p*-Value	Effect Size
Age group	Kruskal–Wallis	χ^2^ = 0.48	0.786	η^2^ = 0.000
Sex	Mann–Whitney U	U = 135,130	0.176	r = 0.049
Educational level	Kruskal–Wallis	χ^2^ = 15.62	**<0.001**	η^2^ = 0.015
Marital status	Mann–Whitney U	U = 120,806	0.750	r = 0.012
Employment status	Kruskal–Wallis	χ^2^ = 3.77	0.152	η^2^ = 0.004
Body weight category	Kruskal–Wallis	χ^2^ = 13.72	**0.003**	η^2^ = 0.014
Chronic disease	Mann–Whitney U	U = 78,338	0.834	r = 0.010
Smoking status	Mann–Whitney U	U = 47,334	**0.007**	r = 0.150
Family history of liver disease	Mann–Whitney U	U = 41,286	**0.001**	r = 0.231

η^2^ = epsilon-squared effect size for Kruskal–Wallis tests. r = rank-biserial correlation for Mann–Whitney U tests. Bold values indicate statistical significance at *p* ≤ 0.05.

**Table 3:** Multivariable linear regression predicting NAFLD knowledge score.

Predictor	B	SE	β	95% CI	*p*-Value
Age (years)	−0.009	0.026	−0.013	−0.061 to 0.042	0.719
Sex (Ref: Male)					
Female	−0.729	0.522	−0.044	−1.754 to 0.296	0.163
Educational Status (Ref: Basic education)					
Secondary education	0.959	1.180	0.047	−1.357 to 3.276	0.417
University education or more	2.783	1.099	0.150	0.627 to 4.940	**0.011**
Occupation (Ref: Not working or retired)					
Governmental employer	−0.258	0.620	−0.015	−1.474 to 0.959	0.678
Private-sector employer	−1.934	0.985	−0.065	−3.867 to −0.001	**0.050**
Smoking Status (Ref: Non-smoker)					
Previous smoker	−0.381	0.959	−0.013	−2.263 to 1.501	0.691
Current smoker	−1.857	0.826	−0.073	−3.477 to −0.237	**0.025**
History of Chronic Disease (Ref: No)					
Yes	0.910	0.714	0.043	−0.491 to 2.311	0.203
Weight Category (Ref: Normal)					
Underweight	−1.335	0.814	−0.056	−2.932 to 0.261	0.101
Overweight	−4.119	1.640	−0.080	−7.337 to −0.901	**0.012**
Obese	−0.365	0.594	−0.021	−1.529 to 0.800	0.539
Family History of liver disease (Ref: No)					
Yes	−0.170	1.024	−0.005	−2.179 to 1.840	0.868

B = Unstandardized beta; SE = Standard error; β = Standardized beta; CI = Confidence interval; Ref = Reference category. Robust standard errors were applied due to detected heteroscedasticity (Breusch–Pagan χ^2^ = 24.22, *p* = 0.029). Significant *p*-values ≤ 0.05 are highlighted in bold.

**Table 4:** Multivariable model diagnostics and assumption checks.

Diagnostic	Result
Variance inflation factor (VIF)	1.02–3.66
Residual normality (Shapiro–Wilk)	W = 0.977, *p* < 0.001
Heteroscedasticity (Breusch–Pagan)	χ^2^ = 24.22, *p* = 0.029
Residual mean	0
Adjusted R^2^	0.022

## Data Availability

The original contributions presented in this study are included in the article/Supplementary Material. Further inquiries can be directed to the corresponding author.

## Supplementary Materials

The following supporting information can be downloaded at: https://www.mdpi.com/article/10.3390/diseases14040139/s1, File S1: Questionnaire in English.

## Abbreviations

The following abbreviations are used in this manuscript:

AIC	Akaike Information Criterion
BIC	Bayesian Information Criterion
CI	Confidence Interval
IQR	Interquartile Range
MASLD	Metabolically Dysfunctional-Associated Steatotic Liver Disease
NAFLD	Nonalcoholic Fatty Liver Disease
NASH	Nonalcoholic Steatohepatitis
Q-Q	Quantile-Quantile
SD	Standard Deviation
SPSS	Statistical Package for the Social Sciences
VIF	Variance Inflation Factor
