# Supplementary material for: Knowledge and Determinants of Nonalcoholic Fatty Liver Disease Among Adults in Northern Border Region, Saudi Arabia: A Cross-Sectional Study
Source: Diseases. 2026 Apr 9;14(4):139. doi: 10.3390/diseases14040139 (PMC13114724; doi:10.3390/diseases14040139)
Supplement: Supplementary file 1 [file diseases-14-00139-s001.zip › diseases-4209749-supplementary.pdf]

<u>Questionnaire in English</u>

Knowledge and Determinants of Nonalcoholic Fatty Liver Disease among Adults in Northern Border Region, Saudi Arabia: A Cross-Sectional Study

A-Personal information

**1.**	**Age**
**2**	**Weight**<br>**Less than 50 kg**<br>**50-69 kg**<br>**70- 90 kg**<br>**More than 90**
**3**	**Height**<br>**Height**<br>**Less than 150 cm**<br>**150-169cm**<br>**170-190 cm**<br>**More than 190**
**4**	**Marital state**<br><br>**Married**<br>**Single**<br>**Divorced**<br>**Widowed**
**5.**	**Educational status**<br><br>**University or higher**<br>**Secondary school**<br>**Preparatory school**<br>**Primary school**<br>**Illiterate**
**6.**	**Occupation**<br>**An employee of a government company**<br>**Employee of a private company**<br>**Not working**
**7.**	**Do you have any chronic health conditions?**<br>1. **None**<br>2. **Diabetes**<br>3. **Hypertension**<br>4. **Congestive heart disease**<br>5. **Others ………**
**8**	**Are you a smoker?**<br>**Never**<br>**Former**<br>**Current**
**9**	**Do you have a Family history of nonalcoholic fatty liver disease?**<br>**Yes**<br>**No**

**10**	**City**<br><br>**Arar**<br>**Rafha**<br>**Turaif**<br>**Al Uwayqilah**<br>**Other ……….**	

B. General knowledge about non-alcoholic fatty liver among participants in the Northern Border Region, Saudi Arabia.

**1**	**Have you heard about non-alcoholic fatty liver disease before?**	• **Yes**<br>• **No**
**2**	**Do you think that fat deposits in the liver are the cause of fatty liver?**	• **Yes**<br>• **No**<br>• **I don't know**
**3**	**Do you think the prevalence of non-alcoholic fatty liver disease in Saudi Arabia is high?**	• **Yes**<br>• **No**<br>• **I don't know**
**4**	**Do you think fatty liver disease can cause dangerous conditions?**	• **Yes**<br>• **No**<br>• **I don't know**
**5**	**Do you think that fatty liver could be due to HCV or HBV?**	• **Yes**<br>• **No**<br>• **I don't know**
**6**	**Do you think that Fatty liver causes liver cirrhosis?**	• **Yes**<br>• **No**<br>• **I don't know**
**7**	**Do you think that Fatty liver can cause liver cancer?**	• **Yes**<br>• **No**<br>• **I don't know**
**8**	**Do you think that fatty liver is related to cardiac problems?**	• **Yes**<br>• **No**<br>• **I don't know**
**9**	**Do you think that a Patient with fatty liver complains of general fatigue?**	• **Yes**<br>• **No**<br>• **I don't know**
**10**	**Do you think that a patient with fatty liver has right hypochondriac pain?**	• **Yes**<br>• **No**<br>• **I don't know**
**11**	**Do you think that a Patient with fatty liver is asymptomatic?**	• **Yes**<br>• **No**<br>• **I don't know**

C. Determinants of developing fatty liver-related knowledge among participants in the Northern Border Region, Saudi Arabia.

**1**	**Do you think that Aging is a risk factor for fatty liver?**	• **Yes**<br>• **No**<br>• **I don't know**
**2**	**Do you think fatty liver disease runs in families?**	• **Yes**<br>• **No**<br>• **I don't know**
**3**	**Do you think Fatty liver is related to obesity?**	• **Yes**<br>• **No**<br>• **I don't know**
**4**	**Do you think that Fatty liver is related to diabetes?**	• **Yes**<br>• **No**<br>• **I don't know**
**5**	**Do you think that Fatty liver is related to hypercholesterolemia?**	• **Yes**<br>• **No**<br>• **I don't know**
**6**	**Do you think that Fatty liver is related to hypertension?**	• **Yes**<br>• **No**<br>• **I don't know**
**7**	**Do you think that Fatty liver is related to overeating?**	• **Yes**<br>• **No**<br>• **I don't know**
**8**	**Do you think that Fatty liver is related to physical inactivity?**	• **Yes**<br>• **No**<br>• **I don't know**
**9**	**Do you think that Fatty liver is related to increased fat intake?**	• **Yes**<br>• **No**<br>• **I don't know**
**10**	**Do you think that Fatty liver is related to increased oil intake?**	• **Yes**<br>• **No**<br>• **I don't know**
**11**	**Do you think that Fatty liver is related to increased sugar intake?**	• **Yes**<br>• **No**<br>• **I don't know**
**12**	**Do you think fatty liver is related to antibiotic overuse?**	• **Yes**<br>• **No**<br>• **I don't know**
**13**	**Do you think that Fatty liver is related to a few sleeping hours?**	• **Yes**<br>• **No**<br>• **I don't know**

D. Prevention and management-related knowledge among participants in the Northern Border Region, Saudi Arabia:

**1**	**Do you think fatty liver disease could be prevented?**	• **Yes**<br>• **No**<br>• **I don't know**

**2**	**Do you think fatty liver disease could be prevented by weight reduction?**	• **Yes**<br>• **No**<br>• **I don’t know**
**3**	**Do you think that fatty liver could be prevented by regular physical activity?**	• **Yes**<br>• **No**<br>• **I don’t know**
**4**	**Do you think that fatty liver could be prevented by a healthy diet?**	• **Yes**<br>• **No**<br>• **I don’t know**
**5**	**Do you think that there is a treatment for fatty liver?**	• **Yes**<br>• **No**<br>• **I don’t know**
**6**	**Do you think that Fatty liver could be easily managed if diagnosed early?**	• **Yes**<br>• **No**<br>• **I don’t know**
